# *Trypanosoma brucei* Polo-like kinase is essential for basal body duplication, kDNA segregation and cytokinesis

**DOI:** 10.1111/j.1365-2958.2007.05866.x

**Published:** 2007-09

**Authors:** Tansy C Hammarton, Susanne Kramer, Laurence Tetley, Michael Boshart, Jeremy C Mottram

**Affiliations:** 1 Infection & Immunity and Wellcome Centre for Molecular Parasitology, University of Glasgow, Biomedical Research Centre 120 University Place, Glasgow G12 8TA, UK; 2 University of Munich (LMU), Department Biology I, Genetics, Maria-Ward-Straße 1a D-80638 Munich, Germany; 3 Infection & Immunity, University of Glasgow Joseph Black Building, Glasgow G12 8QQ, UK

## Abstract

Polo-like kinases (PLKs) are conserved eukaryotic cell cycle regulators, which play multiple roles, particularly during mitosis. The function of *Trypanosoma brucei* PLK was investigated in procyclic and bloodstream-form parasites. In procyclic trypanosomes, RNA interference (RNAi) of *PLK*, or overexpression of TY1-epitope-tagged PLK (PLKty), but not overexpression of a kinase-dead variant, resulted in the accumulation of cells that had divided their nucleus but not their kinetoplast (2N1K cells). Analysis of basal bodies and flagella in these cells suggested the defect in kinetoplast division arose because of an inhibition of basal body duplication, which occurred when PLK expression levels were altered. Additionally, a defect in kDNA replication was observed in the 2N1K cells. However, the 2N1K cells obtained by each approach were not equivalent. Following PLK depletion, the single kinetoplast was predominantly located between the two divided nuclei, while in cells overexpressing PLKty, the kinetoplast was mainly found at the posterior end of the cell, suggesting a role for PLK kinase activity in basal body and kinetoplast migration. *PLK* RNAi in bloodstream trypanosomes also delayed kinetoplast division, and was further observed to inhibit furrow ingression during cytokinesis. Notably, no additional roles were detected for trypanosome PLK in mitosis, setting this protein kinase apart from its counterparts in other eukaryotes.

## Introduction

Polo-like kinases (PLKs) are evolutionarily conserved serine/threonine protein kinases, which play multiple essential cell cycle roles, particularly during mitosis. The founding member of the PLK family, POLO, was identified in *Drosophila melanogaster* (Sunkel and Glover, 1988) as a protein kinase required for spindle assembly during mitosis. Since then, PLKs have been identified in most classes of organisms, with the exception of plants, bacteria and the archaea. *Drosophila*, yeast and kinetoplastids contain a single PLK, while *Caenorhabditis elegans*, *Xenopus*, mice and humans encode four (Plk1–4). Plk1 is the most studied of the mammalian PLKs, and is generally considered to be the functional homologue of *Drosophila* POLO, although a recent study has demonstrated some divergence of function between these PLKs (Pearson *et al*., 2006).

Plk1 functions in multiple cell cycle events such as centrosome duplication/maturation, spindle assembly, the G_2_/M phase and metaphase/anaphase transitions, cytokinesis and the DNA damage response (Dai, 2005), and the multitude of Plk1 substrates reflects this. Additionally, PLKs demonstrate a dynamic localization to different cell structures throughout the cell cycle, consistent with their many functions, although the exact localization pattern is organism dependent (Lee *et al*., 2005). For example, vertebrate Plk1 is found at the centrosomes and kinetochore during late S phase/early G2 phase but relocates to the spindle midzone during the metaphase/anaphase transition and to the midbody during cytokinesis. In contrast, Cdc5 (the POLO homologue in *Saccharomyces cerevisiae*) localizes to the spindle pole bodies in G1 and to the bud neck in late G2 phase, remaining at these sites until late mitosis, and does not translocate to the spindle midzone.

Polo-like kinases have a conserved structure, consisting of an N-terminal kinase domain and a C-terminal polo box domain (PBD) containing one (Plk4) or two (Plk1–3) polo boxes. Both the kinase domain and the PBD of Plk1 have been crystallized (Elia *et al*., 2003b; Kothe *et al*., 2007). The PBD functions as a single modular phosphoserine/threonine-binding domain, recognizing the consensus sequence Ser-[pSer/pThr]-[Pro/X] (Elia *et al.*, 2003a). In the absence of bound substrate, the C-terminus of PLK allosterically inhibits the activity of the kinase domain. Binding of Plk1 through its PBD to a substrate relieves this inhibition, activating the kinase (Elia *et al*., 2003b), an event that is further enhanced by the phosphorylation of the T-loop threonine, T210 (Jang *et al*., 2002; Kelm *et al*., 2002). It is still not clear what upstream kinase carries out this phosphorylation event *in vivo*. PKA, SLK and Plkk1/Stk10 can phosphorylate Plk1 *in vitro*, but it is not apparent if they perform the same function *in vivo* (Qian *et al.*, 1998; Ellinger-Ziegelbauer *et al*., 2000; Kelm *et al*., 2002; Alves *et al*., 2006). Processive and distributive models of substrate phosphorylation by PLK have been proposed (Elia *et al*., 2003b), and PLK activity is controlled by subcellular localization as well as protein abundance and its activation status. PLKs phosphorylate substrates with the consensus sequence [Glu/Asp]-X-[Ser/Thr]-hydrophobic (Nakajima *et al*., 2003), and verified *in vivo* substrates include Cdc25C, cyclin B1, Myt1, Wee1, GRASP65, MKLP1+2, Emi1, claspin and BRCA2 (Lowery *et al.*, 2005).

*Trypanosoma brucei* is a protozoan parasite of considerable medical and economic importance, causing sleeping sickness in humans and Nagana in cattle in sub-Saharan Africa. Some 60 million people are at risk of contracting the disease, and around 300 000 new cases and over 50 000 deaths due to the disease are reported each year. Additionally, the impact of trypanosomiasis infection in livestock is huge, and contributes significantly to poverty of nations affected by the disease. The disease is fatal if left untreated, there is no available vaccine, and current chemotherapies suffer from the problems of toxicity and emerging parasite drug resistance. Hence there is an urgent need to describe and characterize new drug targets. The trypanosome cell cycle is an attractive process to study in the search for novel drug targets, as many aspects of its regulation are unusual compared with other eukaryotes (Hammarton *et al.*, 2003a). The life cycle of *T. brucei* is complex, with the parasite undergoing a series of differentiation steps in two hosts, yielding life cycle stages that differ morphologically, biochemically, structurally and with respect to their replicative status.

The cell cycle of *T. brucei* is regulated, as in other organisms, by the actions of cyclin-dependent and other protein kinases. Unusually though, some eukaryotic checkpoints are not conserved in this organism (Ploubidou *et al*., 1999; Hammarton *et al*., 2003b), and the cell cycles of the procyclic and long slender bloodstream replicative stages are differentially regulated at the molecular level (Hammarton *et al*., 2003b). Furthermore, completion of the genome sequence of *T. brucei* (Berriman *et al*., 2005) has revealed that many conserved cell cycle regulators are apparently lacking, while other regulators, e.g. CDKs and NEKs, are over-represented in this organism, suggesting the operation of novel signal transduction cascades (Naula *et al.*, 2005; Parsons *et al*., 2005). The cell cycle of *T. brucei* is highly complex as the parasite contains a number of single-copy organelles and structures [nucleus, mitochondrion, kinetoplast (containing the mitochondrial DNA), Golgi, basal body/flagellum complex] concentrated in the posterior half of the cell, which must be accurately replicated and segregated in order to generate two viable daughter cells (Hammarton *et al*., 2003a; 2006; McKean, 2003; He, 2007). Additionally, there are two coincident cell cycles – those of the nucleus and kinetoplast. The start of kinetoplast S phase (S_k_) is detected just prior to commencement of nuclear S phase (S_n_), and the kinetoplast divides and segregates before mitosis occurs, resulting in a cell that contains one nucleus and two kinetoplasts (Sherwin and Gull, 1989). Kinetoplast division and segregation is dependent on the prior duplication and segregation of the basal body complex and flagellum (Robinson and Gull, 1991). Mitosis is a closed process in *T. brucei*, and neither chromosome condensation nor nuclear membrane breakdown occurs (Galanti *et al*., 1998; Ogbadoyi *et al*., 2000). Additionally, some *T. brucei* chromosomes are thought to segregate via a novel lateral stacking mechanism (Gull *et al.*, 1998). Following mitosis, in the procyclic form but not the bloodstream long slender form, one of the nuclei migrates to a position between the two segregated kinetoplasts, an action that probably attains the required symmetry for accurate division. Cytokinesis then ensues, but unlike in mammalian cells, furrow ingression is unidirectional, progressing from the anterior end to the posterior end of the cell, along the longitudinal axis, bisecting the cell (Sherwin and Gull, 1989).

Recently, the function of *T. brucei* PLK (Graham *et al.*, 1998) in procyclic trypanosomes was investigated (Kumar and Wang, 2006). This study demonstrated that TbPLK could complement the temperature-sensitive *S. cerevisiae cdc5-1* mutant. RNAi of *PLK* in procyclic *T. brucei* inhibited growth, indicating *PLK* is an essential gene, and analysis of the RNAi mutants suggested a role for PLK in the initiation of cytokinesis in this life cycle stage. We have carried out independent detailed analyses of the function of PLK in procyclic trypanosomes, and demonstrate that PLK is not required for the initiation of cytokinesis *per se.* Rather, downregulation of *PLK* results in an earlier cell cycle defect in basal body duplication, which inhibits kinetoplast segregation and subsequently prevents cytokinesis. Downregulation of *PLK* also causes a delay in kDNA replication. Additionally, we show that overexpression of an active but not kinase-dead version of PLK also inhibits basal body duplication, and by comparison of procyclic RNAi and overexpression mutants, reveal a potential role for PLK in basal body migration. Finally, we extend the functional analysis of PLK to the bloodstream form, and demonstrate a specific role for PLK in cytokinesis furrow ingression in this life cycle stage. However, no role for PLK in trypanosome mitosis was evident, which raises intriguing questions concerning the evolution of both this molecule and the cell cycle in this important pathogen.

## Results

### Downregulation of *PLK* in procyclic *T. brucei* inhibits kinetoplast division

**Fig. 1:**
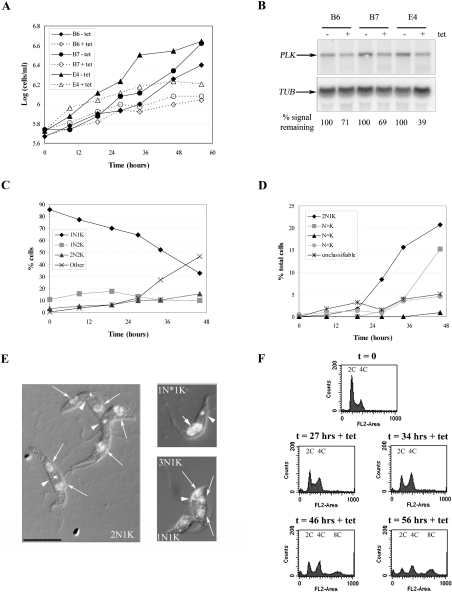
RNAi of *PLK* in procyclic *T. brucei*. A. Representative cumulative growth curves of procyclic *PLK* RNAi clones B6, B7 and E4, passaged to maintain the cell density between 10^6^ and 10^7^ cells ml^−1^, in the presence and absence of 100 ng ml^−1^ tetracycline (tet). B. Northern blot of RNA prepared from *PLK* RNAi clones 24 h post induction, probed with part of the *PLK* ORF (top), and stripped and re-probed with part of the β-tubulin ORF (bottom), as a loading control. The relative intensities of the signal obtained for *PLK* for each clone – and + tetracycline, normalized to the tubulin signal are given. C. DAPI staining of nuclei and kinetoplasts for clone B7, induced with tetracycline. D. Abnormal nucleus-kinetoplast configurations in detail as revealed by DAPI staining for clone B7, induced with tetracycline. E. DAPI/DIC merged images of abnormal cells. Left: 2N1K cells; right top: 1N1K cell with dividing nucleus (1N*1K); right bottom: 3N1K cell. Arrow heads point to kinetoplasts, long arrows to nuclei, and the short arrow to the dividing nucleus. The black bar represents 10 μm. F. Flow cytometry analysis of *PLK* RNAi cells (clone B7) at the time points indicated. The ploidies of the peaks are shown.

To investigate the role of PLK in procyclic trypanosomes, *PLK* was downregulated by RNAi. Three independent *PLK* RNAi clones were analysed. Induction of *PLK* RNAi was achieved by the addition of tetracycline to the culture medium, and resulted in a growth defect visible from 48 h post induction (Fig. 1A), which was accompanied by a 30–60% downregulation of *PLK* mRNA, as demonstrated by Northern blotting (Fig. 1B).

The phenotype of *PLK* RNAi in the three clones was analysed in detail, and as the results obtained were very similar for all clones, only the data for clone B7 are given in subsequent figures unless otherwise stated. Cell cycle progression following *PLK* RNAi induction was monitored by 4,6-diamidino-2-phenylindole (DAPI) staining of DNA and flow cytometry (Fig. 1C–F). DAPI staining (*n* > 200 cells) revealed a significant increase in the number of abnormal cells (Fig. 1C and D), in particular, cells containing two nuclei and one kinetoplast (2N1K). This cell type first appeared at 24 h post induction, and comprised over 20% of the population by 48 h post induction (Fig. 1D and E). From 36 h post induction, significant numbers of cells with >2N began to appear, comprising approximately 15% of the population by 48 h post induction. Many of these cells had only one kinetoplast (Fig. 1E). Very few zoids (0N1K cells; Robinson *et al*., 1995) were present in induced cultures, even at later time points, and examples of 2N2K cells dividing aberrantly to produce 2N1K and 0N1K progeny were not found, indicating the 2N1K cells did not arise from an aberrant cytokinesis event. In contrast, cells with a single kinetoplast and a dividing nucleus were observed (Fig. 1E), arguing that the 2N1K cells arose either because of premature mitosis or because of a delay in kinetoplast replication. However, indications of premature mitosis, as observed previously upon overexpression of the mitotic cyclin, CYC6, in *T. brucei* (T.C. Hammarton and J.C. Mottram, unpubl. data), such as defects in nuclear architecture, or abnormalities in nuclear size, were not observed following *PLK* RNAi (Fig. 1E and data not shown). Taken together, these data demonstrate that downregulation of *PLK* by RNAi inhibits or delays kinetoplast division. Flow cytometry confirmed that replication of nuclear DNA was not inhibited in *PLK* RNAi cell lines, with an increase in the 4C peak (incorporating 2N2K and 2N1K cells) being visible at 34 h post induction, and an 8C peak (>2N cells) appearing at later time points (Fig. 1F).

### Overexpression of kinase-active but not kinase-dead PLKty also inhibits kinetoplast division in procyclic *T. brucei*

**Fig. 2:**
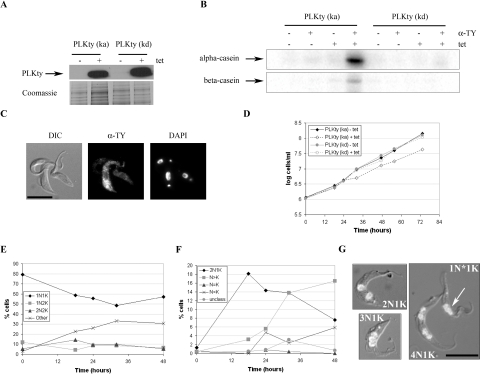
Overexpression of PLKty in procyclic *T. brucei*. A. Western blot analysis of PLKty (ka)- and PLKty (kd)-expressing cells. Cell lines were grown in the presence or absence of tetracycline (tet) (t = 37 h) before cell lysates were prepared for SDS-PAGE and Western blotting with anti-TY antibody (top). Approximately 10^6^ cell equivalents were loaded per lane, and equal loading was confirmed by Coomassie blue staining of a replica gel (bottom). B. Kinase assays. PLKty (ka or kd) was immunoprecipitated from cell lysates with anti-TY antibody, and used in a radioactive kinase assay with alpha- or beta-casein as substrate. C. Immunofluorescence of PLKty (ka)-expressing cells (t = 18 h). Left: DIC image; middle: FITC channel, anti-TY antibody; right: DAPI channel. The black bar represents 10 μm. D. Representative cumulative growth curves of PLKty (ka) and PLKty (kd) cell lines, passaged to maintain the cell density between 10^6^ and 10^7^ cells ml^−1^, in the presence or absence of tetracycline (tet). E. DAPI staining of PLKty (ka) cells, following induction with tetracycline. F. Abnormal cell types in detail, as revealed by DAPI staining for PLKty (ka)-expressing cell lines. Unclass, unclassifiable. G. DAPI/DIC merged images of abnormal cells. The N–K configuration of each cell is given, and the arrow points to a dividing nucleus in a 1N*1K cell. The black bar represents 10 μm.

The effect of overexpressing PLK in procyclic *T. brucei* was investigated. PLK was tagged with the TY-1 epitope tag at its N-terminus. The tag was introduced at the N-terminus in order to avoid a possible deleterious interaction of the epitope tag with the PBD at the C-terminus of the protein. Site-directed mutagenesis was used to generate a kinase-dead version by mutating asparagine 169 in the kinase catalytic domain to alanine (N169A). The PLKty sequences were cloned into pHD675 (Biebinger *et al*., 1997) and transfected into procyclic strain 427 pHD449 (Wirtz and Clayton, 1995), allowing PLKty to be expressed under the control of a tetracycline-inducible promoter. Inducible expression of approximately equivalent levels of PLKty (kinase active, ka) and PLKty (kinase dead, kd) was confirmed by Western blotting of the appropriate cell lysates with the anti-TY (BB2) antibody (Fig. 2A). Kinase assays were performed on PLKty (ka) and PLKty (kd) immunoprecipitated from tetracycline-induced trypanosome cell lysates. PLKty (ka), but not PLKty (kd), showed activity towards alpha-casein, and to a lesser extent, towards beta-casein (Fig. 2B), indicating that the TY-1 epitope tag had not interfered with the kinase activity of PLK, and that the N169A mutation had, as expected, abrogated the kinase activity of PLK. Neither kinase showed activity against myelin basic protein or histone H1 (data not shown).

The subcellular localization of PLKty was investigated by immunofluorescence. PLKty (ka) and PLKty (kd) both displayed a punctate cytoplasmic distribution under a variety of fixation conditions (Fig. 2C and data not shown). No localization to the nucleus, kinetoplast, basal bodies or to the flagellum was observed. Fluorescence was also markedly reduced in cytoskeleton preparations of PLKty-expressing cells, indicating that the majority of the protein is localized to the cytoplasm rather than the cytoskeleton (data not shown). In order to control for possible artefacts caused by the overexpression of PLKty, a procyclic cell line expressing PLKty from the endogenous locus was generated, and expression of PLKty confirmed by Western blot (data not shown). However, the level of expression of PLKty was very low, and was beneath the level of detection by immunofluorescence.

Next, the effect of overexpressing PLKty on the procyclic cell cycle was investigated by monitoring population growth following induction, and by DAPI staining of nuclei and kinetoplasts. Overexpressing PLKty (kd) had no effect on growth (Fig. 2D) or on cell cycle progression (not shown). Overexpression of PLKty (ka) resulted in a reproducible growth defect at 24–36 h post induction, which resolved by 48 h post induction (Fig. 2D). DAPI staining (*n* > 200 cells) showed a significant number of abnormal cells to be present in the culture population from 12 to 18 h post induction, peaking at around 36 h post induction (Fig. 2E), with the number of abnormal cells decreasing thereafter, correlating with the resumption of a normal growth rate. This apparent reversion in phenotype was not, however, due to a downregulation of PLKty (ka) expression as Western blotting of cell lysates revealed PLKty (ka) was still overexpressed 72 h post induction (data not shown), suggesting cells were adapting in another way. Examination of the abnormal cell population revealed it to consist mainly of 2N1K cells (which comprised 18% of the population by 18 h post induction), with >2N1K cells appearing at later time points (Fig. 2F and G). Following the reasoning outlined above for *PLK* RNAi, these 2N1K cells most likely arose as a result of a delay in kinetoplast division, and cells with a single kinetoplast and a dividing nucleus were observed by microscopy (Fig. 2G). Taken together, these data demonstrate that overexpression of PLK kinase activity (but not overexpression of the protein *per se*) inhibits kinetoplast division.

Bloodstream-form cell lines inducibly expressing PLKty were also generated, but PLKty was expressed at very low levels, and no phenotype was detected.

### PLK and kinetoplast positioning

**Fig. 3:**
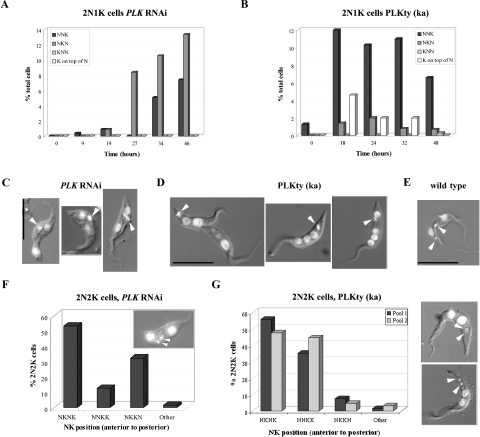
Analysis of 2N1K and 2N2K cells. A and B. The relative positioning of the nuclei and single kinetoplast in 2N1K cells generated by *PLK* RNAi clone B7 (A) and PLKty (ka)-overexpressing (B) cell lines over time following induction. The order of N and K is given from the anterior to the posterior end of the cell. The numbers of cells where the kinetoplast appeared to overlay one of the nuclei are also given. C and D. DAPI/DIC merged images of 2N1K or >2N1K cells from *PLK* RNAi and PLKty (ka)-overexpressing cell lines respectively. Arrowheads point to enlarged/elongated kinetoplasts. The black bars represent 10 μm. E. DAPI/DIC merged image of a wild-type 2N2K cell. Arrowheads point to kinetoplasts. The black bar represents 10 μm. F and G. The arrangement of nuclei and kinetoplasts in 2N2K cells generated by *PLK* RNAi clone B7 (F) and PLKty (ka)-overexpressing (G) cell lines over time. The order of N and K is given from the anterior to the posterior end of the cell. The inset in (F) shows a 2N2K cell with the arrangement NKKN, while the two cell images in (G) show 2N2K cells with a NNKK arrangement. Arrowheads point to kinetoplasts.

The 2N1K cells produced by downregulation of *PLK* by RNAi, and by overexpression of PLKty (ka) in procyclic *T. brucei* were further investigated. The position of the single kinetoplast in 2N1K cells relative to the two nuclei was scored for each cell line. In *PLK* RNAi cell lines, the kinetoplast was located predominantly between the two nuclei (Figs 1E and 3A), whereas in the PLKty (ka)-overexpressing cell line, it was predominantly found at the posterior end of the parasite (Figs 2G and 3B). 2N1K and >2N1K cells with an enlarged and/or elongated kinetoplast were also observed (Fig. 3C and D), suggesting that the kinetoplast divides in these cells (albeit with delayed timing, as the nuclei had already completed one or more mitoses in these cells). To corroborate this, the positions of the two kinetoplasts in 2N2K cells in these cell lines were also investigated. In wild-type procyclic cells, the nuclei and kinetoplasts in a 2N2K cell are arranged N–K–N–K (anterior to posterior, Fig. 3E). In *PLK* RNAi cell lines, however, over 30% of 2N2K cells displayed an N–K–K–N distribution 29 h post induction (*n* = 71; Fig. 3F), while in cell lines overexpressing PLKty (ka), over 35% of 2N2K cells had an N–N–K–K distribution at 18 h post induction (*n* > 65; Fig. 3G). These data are consistent with a model where the level of downregulation of *PLK* or overexpression of PLKty (ka) obtained in this study delays (rather than completely inhibits) kinetoplast division. Downregulation and overexpression of PLK result in the single undivided kinetoplast being positioned differentially relative to the two nuclei, and subsequent kinetoplast division and segregation would account for the abnormal 2N2K cells observed.

### PLK regulates kDNA replication

**Fig. 4:**
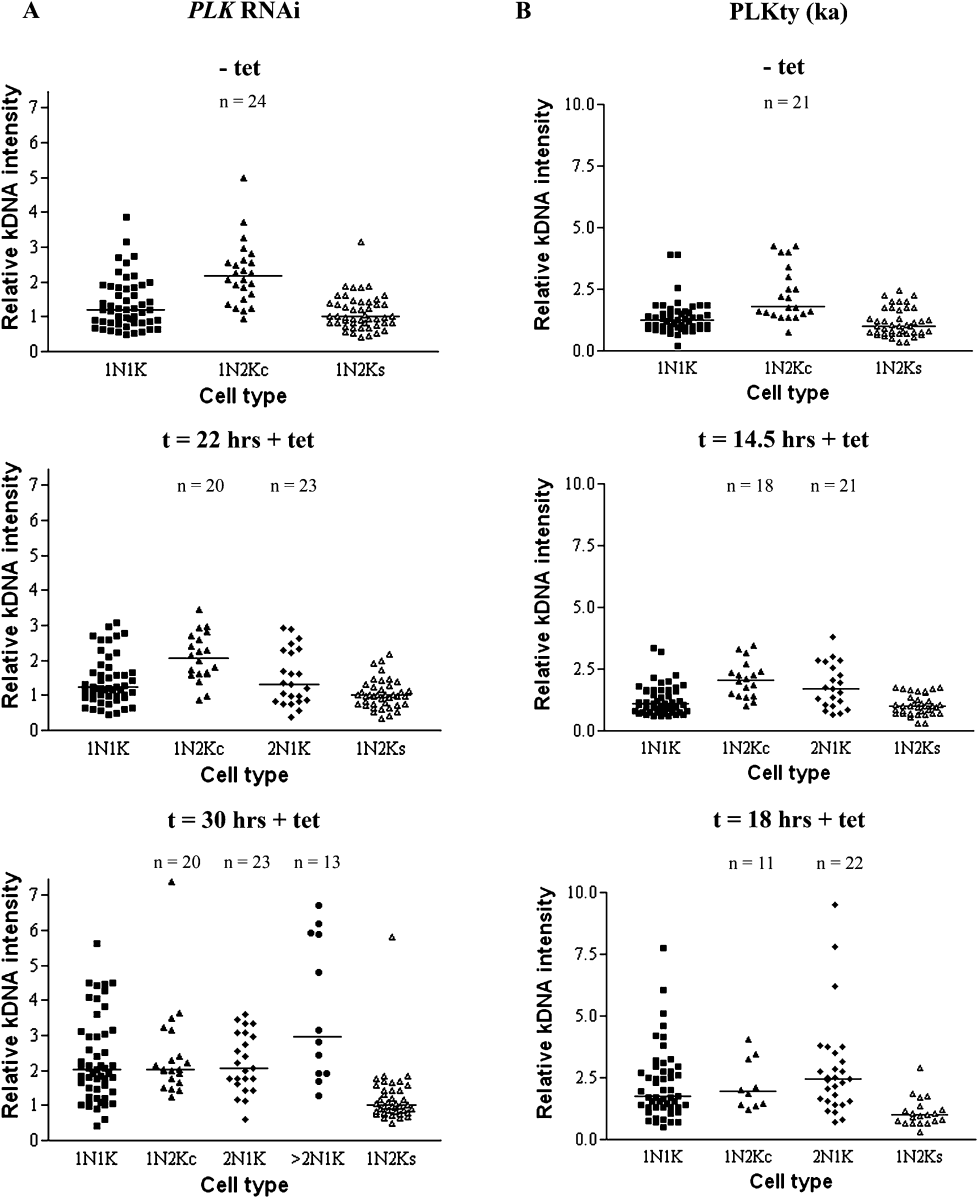
Analysis of kDNA intensity in 2N1K cells. The kDNA intensity was measured in 1N1K, 1N2K, 2N1K and >2N1K cells, and the graphs in (A) and (B) show the distributions obtained for *PLK* RNAi and PLKty (ka)-overexpressing clones, respectively, at the time points indicated. 1N2Kc: combined kDNA intensity values for each kinetoplast; 1N2Ks: kDNA intensity values plotted separately. Horizontal bars indicate the median value of data in each column (also see Table S1). For 1N1K cells, *n* = 50 in all cases; for the other cell types, *n* values are indicated on the graphs.

To investigate whether the single kinetoplasts in the 2N1K cells generated by depletion or overexpression of PLK had replicated their DNA (as would be expected in a post-mitotic cell), images of *PLK* RNAi and PLKty (ka)-expressing cells stained with DAPI were captured and the intensity of the kDNA signal in 1N1K, 1N2K and 2N1K cells was measured over time (Fig. 4 and Table S1). The intensities of the two kDNA networks in 1N2K cells were plotted either combined (1N2Kc) or separately (1N2Ks). As expected, in uninduced (control) populations, the median kDNA intensities of 1N1K cells were slightly higher than the 1N2Ks median intensity values, reflecting that kDNA synthesis had initiated in a subset of the 1N1K population, with the median 1N2Kc intensity values higher still. Following *PLK* RNAi, the kDNA intensities of 2N1K cells were analysed at 22 and 30 h post induction (Fig. 4A). At 22 h post induction, the kDNA intensities of the 2N1K cells were found to be significantly higher than the 1N2Ks values (*W* = 880, *P* = 0.02), but significantly lower than the 1N2Kc values (*W* = 406, *P* = 0.01), indicating that the kinetoplasts in the 2N1K cells had partially, but not fully replicated their DNA at this time point. However, at 30 h post induction, the kDNA intensities of the 2N1K cells were not significantly different from the 1N2Kc values (*W* = 497, *P* = 0.84), indicating that on average, the 2N1K cells had fully replicated their kDNA by this time point. kDNA replication was also able to continue in cells that did not divide (represented by cells with more than two nuclei but only one kinetoplast, >2N1K cells), and the kDNA intensities of the >2N1K cells at 30 h post induction were markedly higher than the 1N2Kc values (*W* = 237, *P* = 0.07). It was also observed that the kDNA intensities of the 1N1K cells increased over time following *PLK* RNAi induction, such that they were no longer significantly lower than the 1N2Kc values (*W* = 1732, *P* = 0.29) at 30 h post induction, reflecting the delayed kinetoplast division.

In PLKty (ka)-expressing cell lines, kDNA intensity was measured 14.5 and 24 h following induction (Fig. 4B). At 14.5 h post induction, the kDNA intensities of the 2N1K cells were significantly higher than the 1N2Ks values (*W* = 813, *P* = 0), and were slightly lower than the 1N2Kc values (*W* = 377, *P* = 0.12), indicating that the kinetoplasts in the 2N1K cells had partially but not fully replicated their DNA at this time point, although the effect was not as dramatic as that observed 22 h following the induction of *PLK* RNAi. At 18 h following induction of PLKty (ka) overexpression, the kDNA intensities of the 2N1K cells were slightly greater than those of the 1N2Kc values, although this difference was not significant (*W* = 570, *P* = 0.22), indicating that the 2N1K cells were able to replicate their kDNA by 18 h post induction, albeit with a delayed time-course. As with the RNAi cells, the kDNA intensities of the 1N1K cells increased over time, such that they were no longer significantly lower than the 1N2Kc values (*W* = 1504, *P* = 0.34) at 24 h post induction, reflecting the delay in kinetoplast division.

### Normally regulated levels of PLK are required for basal body duplication in procyclic *T. brucei*

**Fig. 5:**
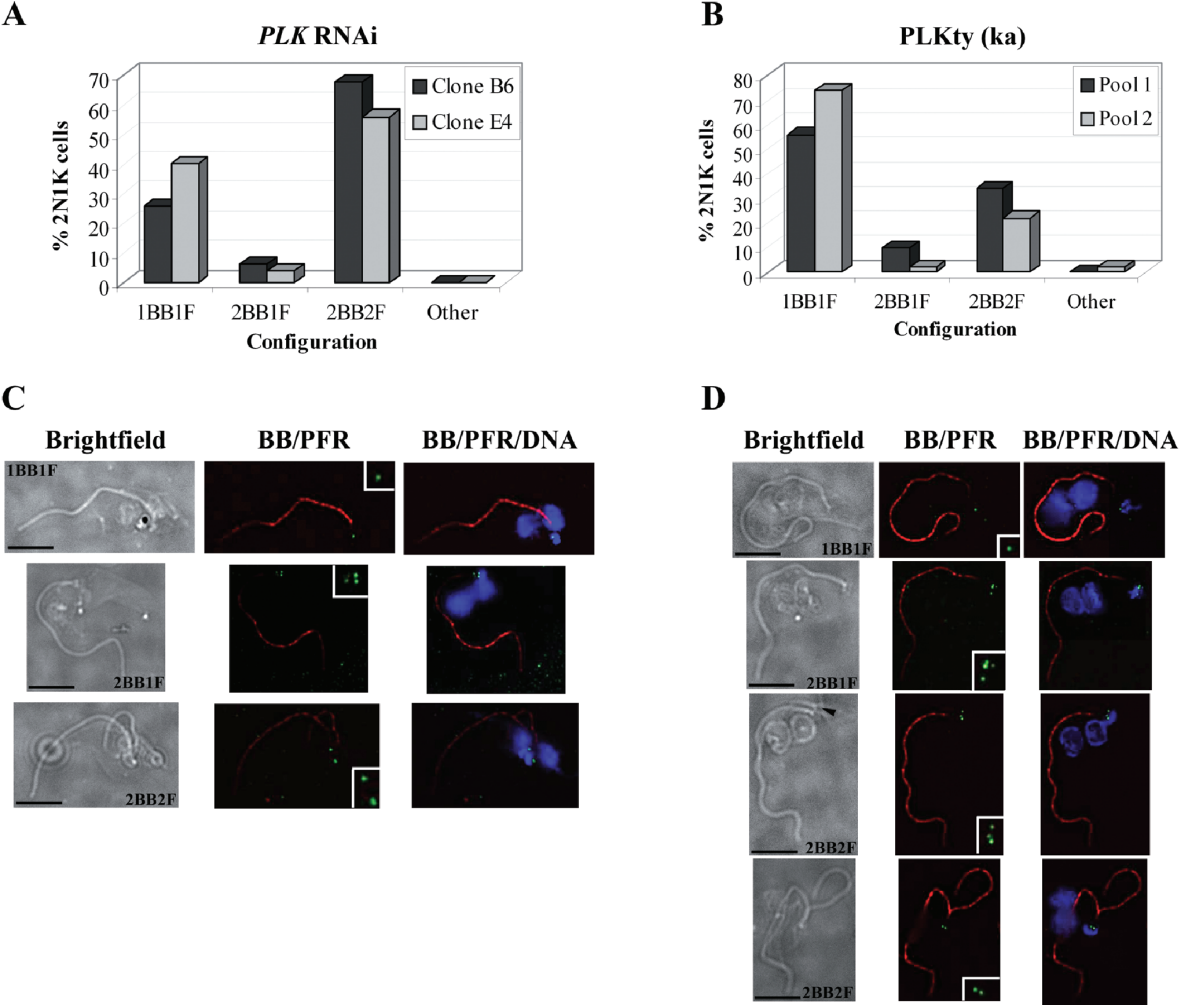
Analysis of basal bodies and flagella in 2N1K cells. The number of basal body pairs (BB) and flagella (F) in cytoskeleton preparations of each 2N1K cell was counted, and the graphs in (A) and (B) show the distributions obtained for *PLK* RNAi and PLKty (ka)-overexpressing cell lines respectively. Example images of the different cell types observed are given in (C) and (D) for *PLK* RNAi and PLKty (ka)-expressing cells respectively. The order of the image panels are (from left to right): brightfield image, anti-basal body staining (BBA4 antibody, green) and anti-PFR staining (L13D6 antibody, red) combined, DAPI (blue)/basal body/PFR merge. The inset panels show the basal bodies at twofold magnification. Note that depending on the orientation of the basal body pair, it is not always possible to see both the pro- and mature basal bodies for a given basal body pair in these images. The number of basal body pairs and flagella per cell is given. The black bars represent 10 μm and the black arrowhead in the third brightfield panel in (D) indicates a new flagellum that presumably has not yet acquired a PFR and therefore does not stain red.

Kinetoplast division and segregation is mediated by basal body and flagellum duplication and segregation (Robinson and Gull, 1991). Early in the cell cycle, trypanosomes contain a pair of basal bodies (a mature basal body subtending the flagellum plus an immature pro-basal body). During G_1_ phase, the pro-basal body matures, nucleates a new flagellum, and two new pro-basal bodies are formed. After kinetoplast S phase, the two basal body pairs pull apart to bring about kinetoplast segregation. To investigate whether the observed defect in kinetoplast division and segregation in *PLK* RNAi and PLKty (ka)-overexpressing cell lines was a downstream effect of defects in basal body duplication and/or segregation, 2N1K cells were further examined to ascertain the number of basal bodies and flagella associated with the single kinetoplast (Fig. 5). Basal bodies were visualized by immunofluorescence by staining cytoskeleton preparations with the BBA4 basal body antibody, while flagella were visualized by a combination of immunofluorescence with the L13D6 (anti-paraflagellar rod, PFR) antibody and brightfield microscopy. For *PLK* RNAi cell lines at 29 h post induction and for cell lines overexpressing PLKty 18 h post induction, up to 68% and 34%, respectively, of the 2N1K cells (*n* = 50) contained two basal body pairs and two flagella, although frequently the daughter flagellum was detached from the cell body and not full length (Fig. 5). However, at these time points, 2N1K cells were also observed which only had one basal body pair/flagellum complex (up to 40% of the 2N1K cell population for RNAi lines, and up to 74% for PLKty-overexpressing cell lines) (Fig. 5). The observation of 2N1K cells with just one basal body pair and one flagellum, as well as the presence of a short daughter flagellum in 2N1K cells with two basal body pairs/flagella, is consistent with a model where basal body (and flagellum) duplication is delayed. This would prevent kinetoplast division and segregation until such time as the basal body/flagellum complex had duplicated and segregated.

**Fig. 6:**
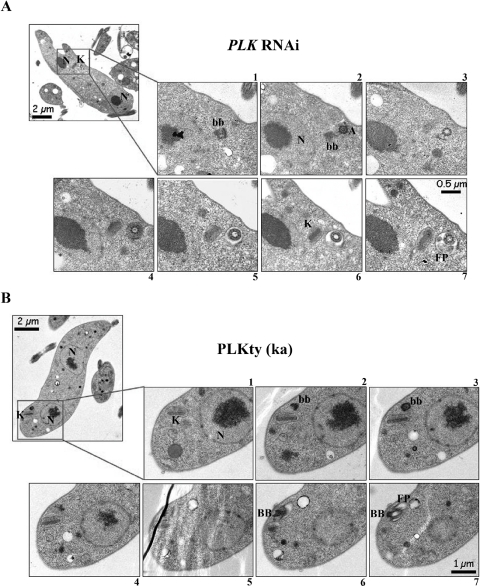
Serial sectioning transmission electron microscopy of 2N1K cells. An example of a 2N1K cell with just one basal body pair and one flagellum is given for *PLK* RNAi (A) and PLKty (ka)-expressing (B) cell lines. Top left panels show a low-magnification image [1840× (A) and 2200× (B)] to illustrate the 2N1K configuration and the lower seven panels are serial higher-power images [6300× (A) and 5000× (B)] of the kinetoplast region. Numbers indicate the order of the serial sections. Key structures in the cell are indicated with letters. N, nucleus; K, kinetoplast; bb, pro-basal body; BB, mature basal body; A, flagellar axoneme; FP, flagellar pocket.

To confirm the delay in basal body duplication, and to exclude the possibility that changes in PLK expression levels had led to a decrease in the protein recognized by the BBA4 antibody, *PLK* RNAi cells and PLKty (ka)-overexpressing cells were examined by serial sectioning transmission electron microscopy (Fig. 6), which confirmed the presence of 2N1K cells with only one basal body pair.

### PLK is essential for cytokinesis furrow ingression in bloodstream-form *T. brucei*

**Fig. 7:**
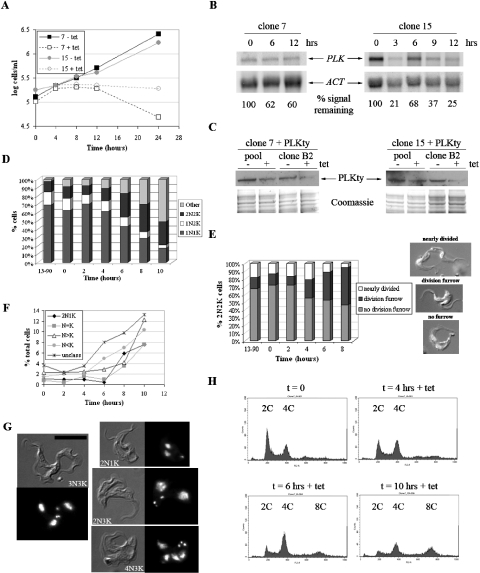
RNAi of *PLK* in bloodstream-form *T. brucei*. A. Representative cumulative growth curves of bloodstream *PLK* RNAi clones 7 and 15, passaged to maintain the cell density between 10^5^ and 10^6^ cells ml^−1^, in the presence and absence of 1 μg ml^−1^ tetracycline (tet). B. Northern blot of RNA prepared from *PLK* RNAi clones up to 12 h post induction. The blots were probed with part of the *PLK* ORF (top), stripped and re-probed with the actin ORF (bottom), as a loading control. The relative intensities of the signals obtained for *PLK* for each clone over time, normalized to the actin signals, are given. C. Western blot analysis of *PLK* RNAi cell lines where one *PLK* allele has been replaced with *PLKty*. Data for one pool and one clone derived from each original clone are shown. Cell lines were grown in the presence or absence of tetracycline (tet) for 6 h before cell lysates were prepared for SDS-PAGE and Western blotting with anti-TY antibody (top). Approximately 1.5 × 10^6^ cell equivalents were loaded per lane, and equal loading was confirmed by Coomassie blue staining of a replica gel (bottom). D. DAPI staining of nuclei and kinetoplasts for clone 7 and the RNAi host cell line (13–90), induced with tetracycline. E. Classification of 2N2K cells according to the stage of cytokinesis (clone 7 and 13–90). Example images of cells in the different stages of cytokinesis are given. F. Abnormal nucleus-kinetoplast configurations in detail as revealed by DAPI staining for clone 7, induced with tetracycline. Unclass, unclassifiable. G. DIC and DAPI images of abnormal cells. The black bar represents 10 μm. H. Flow cytometry analysis of *PLK* RNAi cells (clone 7) at the time points indicated. The ploidies of the peaks are shown.

RNAi was next used to investigate the function of PLK in the bloodstream form of *T. brucei*. *PLK* RNAi was induced in two independent clones by the addition of tetracycline to the culture medium and resulted in a rapid growth arrest (visible from 4 h post induction) (Fig. 7A) that was accompanied by a significant decrease (up to 80% reduction) in *PLK* mRNA as determined by Northern blot analysis (Fig. 7B). To confirm that PLK protein levels also decreased following induction of the RNAi, one of the *PLK* alleles in each of the *PLK* RNAi clones was replaced with a modified *PLK* gene encoding PLKty under the control of the endogenous promoter. This allowed the detection of PLK in the RNAi cell lines without the need for a specific anti-PLK antibody. Western blot analysis of *PLK* RNAi: *PLKty* pools and clones derived from each original RNAi clone demonstrated a decrease (estimated 50–75% reduction) in PLKty protein levels 6 h following induction (Fig. 7C). A similar approach to confirm protein knockdown following RNAi induction in procyclic cells was, however, unsuccessful as *PLK* RNAi: *PLKty* procyclic cell lines could not be generated.

Cell cycle progression in bloodstream-form RNAi clones was monitored by DAPI staining of DNA and flow cytometry (Fig. 7D–H). The phenotypes observed for both bloodstream clones were very similar, and therefore, for these and subsequent experiments, only the data for clone 7 are shown. A significant increase in post-mitotic (2N2K) cells was observed over time by DAPI staining (*n* > 400 cells), with these cells comprising 35% at 8 h post induction (Fig. 7D). The accumulation of post-mitotic cells suggested that cytokinesis was impaired by *PLK* RNAi, and the 2N2K cells were further examined to determine the stage at which cytokinesis was inhibited. By combining DIC and fluorescence microscopy, it was demonstrated that the proportion of furrowing 2N2K cells increased over time, with nearly 50% of 2N2K cells (*n* > 200) at 8 h post induction having a furrow compared with less than 15% at time zero (Fig. 7E). The proportion of 2N2K cells which had completed furrow ingression but not abscission also decreased over time (from 13% at t = 0 to 5% at t = 8 h). These data strongly indicate that furrow ingression is impaired when PLK is downregulated in bloodstream trypanosomes.

*PLK* RNAi also resulted in the appearance of abnormal cell types over time (Fig. 7F and G), with 50% cells having abnormal nucleus/kinetoplast configurations at 10 h post induction. Within the abnormal cell population, no one particular cell type accumulated, but cells with multiple nuclei and/or kinetoplasts were observed (Fig. 7F and G and Fig. S2). Some of the abnormal cells contained furrows, and examples of furrowing 2N2K cells with dividing nuclei and kinetoplasts were also observed, suggesting that the defect in cytokinesis was permitting the re-replication of DNA in the absence of completion of cell division, thereby deregulating the cell cycle. Flow cytometry revealed an increase in cells with 4C DNA content and the appearance of tetraploid cells from 6 h post induction, which was in agreement with the DAPI data (Fig. 7H).

### Downregulation of *PLK* affects kinetoplast division in bloodstream-form trypanosomes

**Fig. 8:**
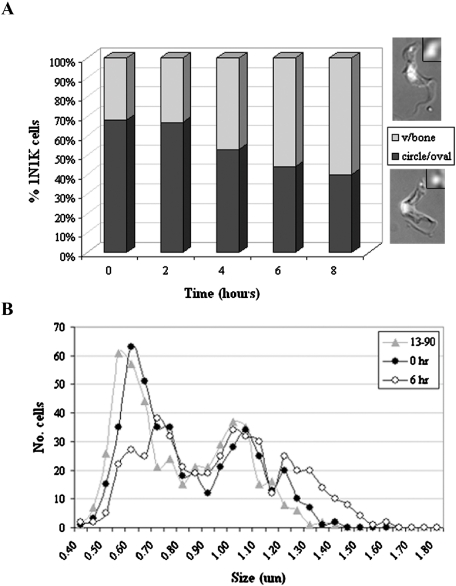
Analysis of kinetoplast division in bloodstream-form *PLK* RNAi cells. A. Distribution of non-dividing (circle/oval shaped, lower cell image) and dividing (v/bone shaped, upper cell image) kinetoplasts in 1N1K cells over time following induction of *PLK* RNAi. B. Distribution of kinetoplast length in the RNAi host cell line (13–90), and *PLK* RNAi clone 7 at time zero and at 6 h post induction. Measurements were rounded to the nearest 0.05 μm.

Following induction of *PLK* RNAi in bloodstream-form cell lines, an increase in the percentage of 1N1K cells with a dividing kinetoplast (either v-shaped or bone-shaped; Helms *et al*., 2006) was also observed by DAPI staining (Fig. 8A). The length of the kinetoplast along its longest dimension was measured in 1N1K cells (*n*> 400) following induction of *PLK* RNAi (Fig. 8B). In the 13–90 host cell line, two major peaks were observed when the number of cells was plotted against kinetoplast length, representing the undivided kinetoplast (circular/oval-shaped, first peak) and dividing kinetoplast (v- or bone-shaped, second peak). Following induction of *PLK* RNAi, kinetoplast length increased significantly compared with the uninduced or host cell line controls (Σ χ^2^ = 303.6 and 755.6, respectively, at 6 h post induction, d.f. = 15, *P* < 0.001). More cells had longer kinetoplasts (larger second peak), and the average kinetoplast length increased (host cell line: 814 nm; uninduced RNAi cell line: 847 nm; induced RNAi cell line: 970 nm), suggesting a delay in the latter stages of kinetoplast division, which probably resulted in the production of the 2N1K cells observed by DAPI staining (Fig. 7F and G). Additionally, there was a significant (albeit smaller) difference in kinetoplast length between uninduced RNAi cells and the 13–90 host cell line (Σ χ^2^ = 75.8, d.f. = 15, *P* < 0.001), probably due to a small amount of leaky expression from the RNAi construct, which correlates with the DAPI data (Fig. 7D), where the uninduced RNAi cell population contained more abnormal cells than the 13–90 control.

## Discussion

In this study, we have demonstrated essential roles for *T. brucei* PLK (TbPLK) in basal body duplication in the procyclic life cycle stage and in furrow ingression during cytokinesis in the bloodstream form, but we did not find a role for TbPLK in mitotic regulation. However, a mitotic role for TbPLK cannot completely be ruled out given that a *PLK* null mutant has not yet been generated, but the absence of any effect on mitosis following RNAi of *PLK* or overexpression of PLK, in this work or a previous study (Kumar and Wang, 2006), is compelling. This is particularly remarkable considering that TbPLK is apparently able to carry out mitotic functions in yeast (Kumar and Wang, 2006), and may suggest that components of mitotic pathways in trypanosomes have evolved to regulate mitosis in the absence of PLK, or that another protein kinase is able to complement mitotic functions of PLK when TbPLK is depleted. Even more intriguing is that TbPLK contains amino acid substitutions at three conserved residues found to be critical for binding to mitotic ligands (W561F, H712K and K714E, equivalent to W414, H538 and K540 in human Plk1) (Lee and Erikson, 1997; Elia *et al*., 2003b), and it is hoped that future structural studies may offer an explanation for how TbPLK can interact with mitotic substrates in yeast.

The requirement of TbPLK for basal body duplication in procyclic trypanosomes is reminiscent of the functions of Plks in centriole duplication in human and *Drosophila* cells. Human Plk2 kinase activity is required for centriole duplication (Warnke *et al*., 2004), while depletion of Plk4 from humans or *Drosophila* prevents centriole duplication, and overexpression of Plk4 causes centriole amplification (Bettencourt-Dias *et al*., 2005; Habedanck *et al*., 2005). In humans, depletion of Plk4 leads to errors in mitosis, while *Drosophila* cells are able to undergo mitosis, but are unable to form basal bodies or flagella. *T. brucei* contains multiple microtubule-organizing centres (MTOCs), but no centrioles, although the basal bodies are based on a canonical centriolar structure. As in other eukaryotes, γ-tubulin localizes to the trypanosome MTOCs, and is required for central pair nucleation of the basal body (McKean *et al*., 2003). δ-Tubulin is also required for formation of correctly structured basal bodies in *T. brucei* (Gadelha *et al*., 2006), and TbCentrin1 and TbCentrin2 are essential for basal body duplication (He *et al.*, 2005). Two other proteins, TbLRTP and NRKC, a NIMA-related kinase, have been localized to the basal bodies (Morgan *et al*., 2005; Pradel *et al*., 2006). TbLRTP negativelyregulates basal body duplication and flagella biogenesis, as its overexpression reduces the number of basal bodies per cell and inhibits new flagellum assembly, but its depletion by RNAi results in additional basal bodies, axonemes and PFRs being formed (Morgan *et al*., 2005). NRKC, on the other hand, may activate the separation of the old and newly matured basal bodies early in the cell cycle. RNAi of *NRKC* or overexpression of kinase-dead NRKC in procyclic cells resulted in the accumulation of cells with two basal body pairs, while overexpression of active kinase generated multinucleate cells with a single kinetoplast associated with a single flagellum and multiple immature basal bodies (Pradel *et al*., 2006).

Unlike TbLRTP and NRKC, where depletion and overexpression of the proteins have differential effects on basal body duplication, any changes to the expression levels of TbPLK appear to inhibit this process. In particular, very few cells with supernumerary basal bodies were observed following modulation of PLK expression. This suggests the existence of a feedback loop to control the activity of PLK. Downregulation of PLK delays basal body duplication, arguing that PLK is required for this process. However, an increase in active, but not kinase-dead PLK, also delays basal body duplication. Nothing is known at present about the expression of PLK through the trypanosome cell cycle (effective cell cycle synchronization of *T. brucei* has not yet been achieved), but a model can be envisaged where a certain level of PLK activity is required for basal body duplication early on in the cell cycle. Once this process is complete and/or PLK activity exceeds a threshold, a feedback pathway is invoked. This pathway might downregulate PLK activity, divert its basal body duplication-stimulating activity, or activate a negative regulator of basal body duplication such as TbLRTP, perhaps as a way of ensuring basal bodies are only duplicated once per cell cycle. Overexpression of PLK kinase activity would therefore activate the feedback pathway and prevent basal body duplication.

The data in this study demonstrate that kDNA replication is delayed in 2N1K cells at early time points following induction of *PLK* RNAi or overexpression of PLKty (ka). This delay appears to be temporary, as at later time points, the kinetoplasts in 2N1K and >2N1K cells were able to replicate and re-replicate their DNA. It is not clear at present whether the defect in kDNA replication is linked to the delay in basal body duplication in these cell lines or whether PLK operates at two separate points at the G_1_/S phase boundary. To date, kDNA replication has not been analysed in other *T. brucei* cell lines where basal body duplication is perturbed. However, it is known that in this organism, kDNA replication begins prior to basal body duplication (Woodward and Gull, 1990) and continues during kinetoplast segregation (Ogbadoyi *et al.*, 2003). kDNA replication is also essential for the attachment of the mitochondrial genome to the tripartite attachment complex following its replication (Ogbadoyi *et al.*, 2003). The relationship between basal body duplication and kDNA replication at the G_1_/S phase boundary clearly warrants further study.

This study also hints at an intriguing role for PLK in basal body migration to the posterior end of the cell in procyclic trypanosomes. Following kDNA replication, the kinetoplast is divided and segregated by the movement of the basal bodies. The newly formed basal body/flagellum complex moves to the posterior end of the cell, taking the daughter kinetoplast (to which it is connected via the tripartite attachment complex; Ogbadoyi *et al.*, 2003) with it. Following mitosis, one of the nuclei moves into the space between the segregated kinetoplasts. In the 2N1K cells (N–K–N) obtained via *PLK* RNAi, it appears the old basal body/flagellum complex and undivided kinetoplast do not move significantly, but that the nuclei migrate as normal following mitosis. However, following PLKty (ka) overexpression, the single kinetoplast is located posterior to the two nuclei, suggesting that the old basal body/flagellum complex has migrated towards the posterior end of the cell, taking the undivided kinetoplast with it. A role in regulating basal body migration, to our knowledge, has not been documented for a PLK before. Plk1 is required for maturation of centrosomes (Dai, 2005), but centrosome separation depends on the kinesin Eg2 (Lane and Nigg, 1996) as well as phosphorylation of NDEL1 by Aurora-A kinase (Mori *et al*., 2006). The Rho-associated protein kinase, ROCK, was demonstrated to regulate centrosome positioning in HeLa cells (Chevrier *et al*., 2002), but neither a ROCK homologue nor Rho GTPases are identifiable in *T. brucei*. However, basal body migration in *T. brucei* depends on the microtubule cytoskeleton (Robinson and Gull, 1991), and *Drosophila* Polo kinase has been shown to phosphorylate microtubule-associated proteins (Glover, 2005), raising the possibility that TbPLK could control basal body migration through phosphorylation of cytoskeletal proteins. The discovery of *in vivo* substrates for TbPLK should shed more light on its potential basal body migratory role.

The roles for TbPLK in procyclic *T. brucei* proposed in this study differ from that reported by Kumar and Wang (2006). They concluded that PLK is required for the initiation of cytokinesis, as they saw the accumulation of cells with multiple nuclei, kinetoplasts, flagella and basal bodies from 2 days post induction of *PLK* RNAi. Our analysis has studied the effects of downregulating PLK at earlier time points, and reveals that the first event to be disrupted is not cytokinesis, but the duplication of the basal bodies. It is known that basal body duplication and segregation is essential for cytokinesis to occur (Ploubidou *et al*., 1999; He *et al.*, 2005) (indeed, the 2N1K cells generated here appeared to be unable to divide), and therefore, although an additional role for TbPLK in cytokinesis cannot be ruled out, the inhibition of cytokinesis observed is likely to be a downstream effect of the basal body duplication defect. As basal body duplication in *PLK* RNAi cell lines is only delayed and not completely blocked (presumably because PLK is only partially knocked down, see Fig. 1B), cells with multiple organelles accumulate at later time points. Significantly, Kumar and Wang detected 14% 2N1K cells (many of which appeared to have an N–K–N arrangement) 1 day after RNAi induction, providing further evidence for the conclusions reached here.

One discrepancy between this work and that of Kumar and Wang is in the subcellular localization of PLK. In this study, PLKty (ka and kd) was visualized as discrete cytoplasmic puncta throughout the cell cycle. No localization to the nucleus, kinetoplast or any flagellar structures was observed in this study, and although it is possible that this cytoplasmic localization was an artefact of the overexpression of PLKty, no fraction of the signal was ever localized to the basal bodies. This is not, however, inconsistent with the proposed role for PLK in basal body duplication as PLK may act upstream and phosphorylate other regulators, which in turn localize to the basal bodies. Kumar and Wang used a C-terminal HA-tagged version of TbPLK to localize PLK to the anterior tip of the flagellum and FAZ. One explanation for the differing localizations may lie in the location of the epitope tag. In the Kumar and Wang study, PLK was tagged at the C-terminus, just two amino acids downstream of the PBD, a region that is likely to be crucial for subcellular localization. Hence it is possible that the 3HA tag interfered with the functioning of the PBD, a risk that is minimized with our N-terminal TY1 tagging strategy. We have demonstrated that PLKty is active; it is not known whether TbPLK-3HA is also active, but no phenotype was detected upon overexpressing it until 5 days post induction. In this study, a significant, but transitory phenotype was observed between 12 and 48 h post induction, and given that trypanosomes appear to be able to adapt to the effects of overexpressed PLK, it is possible that the localization of PLK-3HA at 5 days post induction does not accurately reflect the localization of endogenous PLK.

The analysis of TbPLK has been extended to the bloodstream form in this study. Here, following *PLK* RNAi, kinetoplast division was delayed (although not completely blocked) and 2N1K cells were observed by DAPI staining, suggesting that PLK may also play a role in basal body duplication in this life cycle stage. However, PLK depletion also inhibited furrow ingression during cytokinesis. This phenotype was very similar to that obtained upon depletion of MOB1 (Hammarton *et al*., 2005) in this life cycle stage, and similarly resulted in a deregulation in the cell cycle, as the delay in furrowing allowed cells to re-replicate their nuclei and kinetoplasts. This may suggest that MOB1 and PLK function in the same regulatory pathway. In a manner similar to the localization of PLKty in the procyclic form, MOB1ty was found to localize to the cytoplasm of bloodstream-form trypanosomes, with no detection of the protein at the furrow, again suggesting that MOB1 may be an upstream regulator of cytokinesis, rather than a direct effector of the process. In *S. cerevisiae*, MOB1 and PLK function in the mitotic exit network, allowing exit from mitosis and entry into cytokinesis. Although the mitotic functions of these proteins have apparently not been retained in *T. brucei*, it will be interesting to discover if they instead both function in the same cytokinesis pathway. The mechanism of cytokinesis in trypanosomes is distinct from that seen in mammalian cells, with the furrow ingressing unidirectionally from the anterior to the posterior end of the cell, rather than relying on a contractile actomyosin ring. Actin has not so far been linked to trypanosome cytokinesis (García-Salcedo *et al*., 2004), and nothing is known about the function of myosins in *T. brucei*. Additionally, Rho GTPases, which play vital roles in regulating cytokinesis in mammalian cells, appear to be absent from trypanosomes. Depletion of many trypanosome proteins have been shown to inhibit cytokinesis initiation, but to date, only MOB1 and TRACK (Rothberg *et al*., 2006) have been shown to have a specific function in furrow ingression in *T. brucei*, and the identification of a third regulator is an important step forward in our understanding of this process.

Finally, the differences in function of TbPLK compared with mammalian PLKs may reflect differences in amino acid sequence and tertiary structure, which would suggest that it has potential as a drug target. For example, within the kinase domain, TbPLK shares only 48% identity and 64% similarity at the amino acid level with human Plk1. Recently, homology modelling of the Plk1 kinase domain led to the discovery of a group of potent Plk1 inhibitors containing a benathiazole *N*-oxide core structure (McInnes *et al*., 2006). Modelling predicted that the inhibitor makes several contacts with the aromatic ring of Phe183 within the active site of Plk1; the corresponding residue in TbPLK is Met171, suggesting that the active site of the parasite enzyme may be sufficiently different to allow selective inhibition. Plk1 is overexpressed in human tumours, an event that correlates with poor prognosis (Eckerdt *et al.*, 2005). Currently, considerable effort is being expended to obtain Plk1 inhibitors for use in cancer therapy (Strebhardt and Ullrich, 2006), and it may be possible to exploit these efforts in the future to generate new, much needed antiparasitic agents.

## Experimental procedures

### Recombinant DNA techniques

Standard DNA techniques were performed as described in Sambrook *et al.* (1989). Plasmid isolation from *Escherichia coli*, and DNA extraction from agarose gels used Qiaprep and Qiaex kits from Qiagen respectively. DNA sequencing was performed by the University of Munich sequencing service, using an ABI automatic sequencer.

### Transfection of *T. brucei*

Culture and transfection of *T. brucei* was carried out as described previously (Vassella *et al*., 2001; Hammarton *et al*., 2003b; Burkard *et al.*, 2007).

### PLK RNAi

For RNAi, the procyclic 427 pLew13 pLew29 and the bloodstream-form 427 pLew13 pLew90 cell lines (Wirtz *et al*., 1999) were transfected (Hammarton *et al*., 2003b) with plasmid p2T7-PLK-Nterminus. To generate p2T7-PLK-Nterminus, the *GFP* insert in plasmid p2T7_ti_:GFP (LaCount *et al*., 2000) was removed by digestion with BamHI and XbaI, and replaced with the first 306 nucleotides of the *T. brucei PLK* open reading frame (ORF), amplified by the polymerase chain reaction (PCR) from genomic DNA of strain MiTat1.2. blast analysis and RNAit software (http://trypanofan.path.cam.ac.uk/software/RNAit.html) confirmed this partial *PLK* sequence to be unique in the trypanosome genome, and therefore unlikely to cause the downregulation of any other mRNA. Independent trypanosome clones were generated by limiting dilution cloning. RNAi was induced as described previously (Hammarton *et al*., 2003b).

To confirm that PLK protein levels were downregulated following induction of the RNAi, one of the *PLK* alleles in bloodstream *PLK* RNAi clones was replaced by a TY1-tagged copy of *PLK* using the *in vivo* epitope tagging strategy described by Shen *et al*. (2001). Briefly, clones were transfected with 10 μg of PCR product amplified from pBS-BLA/BB2 using the oligonucleotides OL1953 (5′-ACATTTGAAGCACTGCCGTCAGCGTGTGTTGTATTGCGTTGGTTTGATTATAACGGAGGGGGGGGGAAAAAAAAAAAAGAATGGCCAAGCCTTTGTCTCAAG-3′) and OL1954 (5′-GAACCCTCACGAAAGTCCGGCCTGTCATTTGGAGGGCGCCGCGAACTCGACGGCGTTTCACACGTCTCAGCGGTTGCGTGGTCAAGTGGATCCTGGTTAGTATG-3′), which introduced 80 nucleotides of the 5′ untranslated region and 5′ end of the *PLK* ORF, respectively, at the ends of the PCR product to allow integration into the native locus generating a *TY1–PLK* fusion. The RNAi response was induced in pools and clones obtained from these transfections following selection with 10 μg l^−1^ blasticidin. Growth rate and morphological analyses were used to confirm that these transfectants still responded as expected to the addition of tetracycline to the medium. Cell lysates were prepared 6 h post induction for analysis by Western blotting with the anti-TY (BB2) antibody (see below).

### Northern blotting

Twenty micrograms of total RNA [prepared from cells using the TRIZOL reagent (Invitrogen)] was electrophoresed on a 1.2% agarose-formaldehyde gel alongside 2 μg of RNA Millennium size markers (Ambion), and visualized under UV light to confirm equal loading. RNA was capillary transferred to Hybond N membrane (Amersham) in 20× SSC for 5 h and UV-cross-linked to the membrane, with transfer confirmed by methylene blue staining. Following destaining of the membrane in 0.2× SSC, 1% SDS, the membrane was pre-hybridized in Church Gilbert's buffer (Church and Gilbert, 1984) at 65°C for 1 h. DNA probes were radiolabelled with [α-^32^P]-dATP using the Prime-It kit (Stratagene), denatured by heating to 95°C and hybridized to the membrane in Church Gilbert's buffer at 65°C overnight. Following extensive washing in SSC/SDS buffer, the membrane was exposed to a phosphorimager plate overnight and scanned on a Typhoon phosphorimager. A 0.9 kb BamHI–SacI fragment of pGL1278 comprising the 3′ end of the *PLK* ORF, or a 2.28 kb PCR product (amplified using oligonucleotides *PLK* forward: 5′-CTGCGGATCCATGCACGCAACCGCTGAGAC-3′ and *PLK* reverse 5′-TCTTTGGTGTAACGAAGCCG-3′) comprising most of the *PLK* ORF was used as a *PLK* probe. The entire actin ORF was used as the actin probe, while a 635 bp PCR product (amplified using oligonucleotides 5′-GCCCCGACAACTTCATCTTTGGA-3′ and 5′-TTTCGCATCGAACATCTGCTGCG-3′) was used as the tubulin probe.

### PLKty overexpression

To facilitate detection of PLK, a TY-1 epitope tag was fused to the N-terminus of the protein. This was achieved by PCR-amplifying *PLK* from MiTat1.2 genomic DNA using the oligos 5′-TCACAAGCTTATGGGTGAGGTCCATACTAACCAGGACCCACTTGACCACGCAACCGCTGAGACG-3′ (incorporating a HindIII restriction site, the methionine start codon, the TY-1 epitope tag coding sequence and the coding sequence for the N-terminus of *PLK*) and 5′-ACGTGGATCCCTAAATATCACCGTTTTGTATGAG-3′ (incorporating a BamHI restriction site). The resultant PCR product was digested with HindIII and BamHI, and cloned into pHD675 (Biebinger *et al*., 1997) digested with the same enzymes, generating pGL1278 [PLKty (ka)]. Site-directed mutagenesis was used to mutate N169A, creating a kinase-dead variant, pGL1279 [PLKty (kd)]. Sequencing confirmed the manipulations were successful, and additionally revealed a number of differences compared with the published genome sequence (strain TREU 927) (Table S1). Plasmids pGL1278 and pGL1279 were linearized by NotI digestion and transfected into procyclic and bloodstream-form 427 pHD449 cell lines (Biebinger *et al*., 1997). Expression of PLKty (ka) and PLKty (kd) was induced by the addition of 100 ng l^−1^ (procyclic) or 1 μg l^−1^ (bloodstream) tetracycline to the medium. Cell lysates were prepared for Western blotting by centrifuging at 600 *g* (procyclic cells) or 1500 *g* (bloodstream-form cells) for 10 min, washing once in trypanosome dilution buffer (TDB) (5 mM KCl, 80 mM NaCl, 1 mM MgSO_4_, 20 mM Na_2_HPO_4_, 20 mM glucose, pH 7.4), re-suspending in NuPAGE loading buffer (Invitrogen) and heating to 70°C for 10 min.

### Immunoblotting

Proteins were separated by SDS-PAGE using NuPAGE gels (Invitrogen) and either stained with Coomassie blue to check loading, or transferred to polyvinylidine difluoride membrane (NEN). The See Blue Plus2 pre-stained standard (Invitrogen) was used as a molecular weight marker. Western blots were performed as described previously (Mottram and Grant, 1996) with a 1:20–1:100 dilution of the mouse monoclonal anti-TY-1 (BB2) antibody (Bastin *et al*., 1996) followed by a 1:10 000 dilution of goat anti-mouse IgG horseradish peroxidase-conjugated secondary antibody (Promega). The West-Dura chemiluminescence detection system (Pierce) was used to visualize antigens.

### Immunoprecipitation of TY-tagged proteins and kinase assays

S100 lysates (prepared as described previously; Van Hellemond *et al*., 2000) were incubated with 50 μl of anti-TY antibody or 50 μl of lysis buffer as a negative control for 2 h at 4°C on an end-over-end mixer. Fifty microlitres of prepared protein G beads (0.5 mg l^−1^ in lysis buffer) were added, and the sample mixed for a further 30 min. The beads were extensively washed with lysis buffer and used for kinase assays with histone H1, myelin basic protein, alpha-casein and beta-casein as described previously (Van Hellemond *et al*., 2000).

### Immunofluorescence

Immunofluorescence was carried out as described previously, using either methanol (Hammarton *et al*., 2003b) or paraformaldehyde fixation (Hammarton *et al.*, 2004) or cytoskeleton preparations (Briggs *et al*., 2004). The primary antibodies, BB2 (anti-TY, at 1:20 dilution) (Bastin *et al*., 1996), BBA4 (anti-basal body, at 1:2 dilution) and L13D6 (anti-PFR, at 1:10 dilution) (both kind gifts from Professor Keith Gull, University of Oxford) were used in combination with appropriate Alexa Fluor 488 and 594 conjugated goat anti-mouse secondary antibodies (Molecular Probes, 1:2000). As negative controls, samples were incubated either without primary antibody or without secondary antibody. Nuclear and mitochondrial DNA was stained with DAPI. Following staining, cells were viewed under UV light either on a Zeiss Axioplan microscope with images processed with a Hamamatsu ORCA-ER digital camera or on a DeltaVision RT image restoration microscope system (Olympus IX-71 microscope with a Dual CoolSNAP/Cascade II camera). Image manipulation and deconvolution were carried out using either OpenLab version 15.0.0 software or SoftWorx Imaging software respectively.

### kDNA intensity analysis

To carry out kDNA intensity analyses, images of DAPI-stained cells were acquired at very short camera exposure times (typically < 20 ms) to prevent saturation of the camera and to minimize photobleaching. ImageJ software (http://rsb.info.nih.gov/ij/) was used to measure the integrated density of the kDNA signal(s) in 1N1K, 1N2K and 2N1K cells. 2N2K cells were not included in the analysis, as it was considered possible that cytokinesis defects might result in 2N2K cells starting to re-replicate their DNA, thereby skewing the data. Briefly, the image background was subtracted using a ‘rolling ball’ radius of 200 pixels, the outline of the kinetoplast was traced manually and the integrated density (proportional to DNA content) of the kDNA was obtained. kDNA intensity data were plotted in GraphPadPrism version 3.02, and Mann–Whitney *U*-tests were carried out using Minitab version 13.1. For 1N2K cells, the kDNA intensity values obtained were plotted in combination (1N2Kc) or singly (1N2Ks). Because no defect in mitosis was observed following *PLK* RNAi or overexpression of PLKty, the 1N2Ks and 1N2Kc values give the minimum and maximum kDNA intensity values expected normally before and after replication, respectively, within each population. This allows kDNA intensity values of other cell types (1N1K, 2N1K, >2N1K) to be compared with these baseline values. To facilitate comparisons between different time points, relative kDNA intensity values were computed by expressing each kDNA intensity measurement as a ratio of the 1N2Ks median value for that sample.

### Kinetoplast length analysis

The lengths of kinetoplasts were measured from images of DAPI-stained 1N1K cells using IPLab version 3.9.1 (Scanalytics, Fairfax, USA). Kinetoplast lengths were rounded to the nearest 0.05 μm, and the resulting distributions were compared by χ^2^ goodness of fit analysis.

### Flow cytometry

Analysis of propidium iodide-stained cells by flow cytometry were carried out as described previously (Hammarton *et al*., 2003b).

### Serial sectioning transmission electron microscopy

Trypanosomes were fixed using a modification of Begg *et al.* (1978). Briefly, cells were harvested, fixed for 40 min at 20°C in 2.5% (v/v) glutaraldehyde containing 1% tannic acid in 0.1 M phosphate buffer, and post-fixed in 0.5% osmium tetroxide (w/v) in 0.1 M phosphate buffer, pH 7.0, for 30 min on ice. Following three changes in distilled water (10 min each), specimens were dehydrated via an alcohol series and embedded in Araldite/EMbed 812 resin mix (Electron Microscopy Sciences). Sections (100 nm) were stained with aqueous 2% uranyl acetate and Reynold's lead citrate for 10 and 5 min respectively. Series of 16 sections were viewed on 2 mm × 1 mm slot grids by zero-loss imaging on a LEO 912 AB energy filtering transmission electron microscope.

## Supplementary material

The following supplementary material is available for this article:

Fig. S1 Bloodstream form PLK RNAi cells were stained with DAPI and the number of nuclei and kinetoplasts per cell counted. The tables give details of the configurations of abnormal cell types over time for clones 7 and 15.

Table S1 The sequence of PLK derived from strain MiTat221 was compared to that published for strain TREU 927 GUTat.10.1. Base changes and the corresponding amino acid changes are given.

Table S2 kDNA intensity was measured in 1N1K, 1N2K, 2N1K and > 2N1K cells over time for PLK RNAi cells (clone E4) and PLKty overexpressing cells (pool 1). The median relative kDNA intensity values for each cell type are given (1N2Kc: kDNA intensity values combined; 1N2Ks: kDNA intensity values taken separately).

This material is available as part of the online article from: http://www.blackwell-synergy.com/doi/abs/10.1111/j.1365-2958.2007.05866.x (This link will take you to the article abstract).
