# Supplementary material for: Trypanosoma brucei Polo-like kinase is essential for basal body duplication, kDNA segregation and cytokinesis
Source: Mol Microbiol. 2007 Sep;65(5):1229–48. doi: 10.1111/j.1365-2958.2007.05866.x (PMC2169650; doi:10.1111/j.1365-2958.2007.05866.x)
Supplement: Fig. S1 — Bloodstream form PLK RNAi cells were stained with DAPI and the number of nuclei and kinetoplasts per cell counted. The tables give details of the configurations of abnormal cell types over time for clones 7 and 15. [file mmi0065-1229-SD1.pdf]

Table S1

Median relative kDNA intensity values

***PLK* RNAi cells**

Sample	Cell type	Relative median kDNA intensity
- tet	1N1K	1.20
	1N2Kc	2.17
	1N2Ks	1.00
t = 22 hrs + tet	1N1K	1.22
	1N2Kc	2.06
	2N1K	1.31
	1N2Ks	1.00
t = 30 hrs + tet	1N1K	2.05
	1N2Kc	2.03
	2N1K	2.05
	>2N1K	2.98
	1N2Ks	1.00

PLKty overexpressing cells

Sample	Cell type	Relative median kDNA intensity
- tet	1N1K	1.25
	1N2Kc	1.78
	1N2Ks	1.00
t = 14.5 hrs + tet	1N1K	1.08
	1N2Kc	2.06
	2N1K	1.69
	1N2Ks	1.00
t = 18 hrs + tet	1N1K	1.75
	1N2Kc	1.93
	2N1K	2.43
	1N2Ks	1.00

Table S2

Changes in sequence of *PLK* from MiTat221 compared to the genome strain TREU 927 GUTat 10.1.

Base	TREU 927 GUTat.10.1	MiTat221	Mutation
960	G	A	Silent
1245	CAA CAA CAA CAA CAA	Deleted	Deletion of NNNNN
1282	AAC AAC AAC AAC	GCC ATA AAC CCC	NNNN to AINP
1344	A	G	Silent
1438	G	A	V to A
1485	T	C	Silent
1515	T	A	Silent
1582	C	G	P to A
1830	T	C	Silent
2025	C	T	Silent
2142	A	G	silent

Figure S1 - Abnormal cells for bloodstream form *PLK* RNAi cell lines

Clone 7 — t=6 hrs + tet — total cells counted = 514, total abnormal cells = 87 (16.9%), of which 30 (5.8%) were unclassifiable

number of nuclei	number of kinetoplasts						
	0	1	2	3	4	5	6
0		3	1				
1	9						
2	3	2		7	7		1
3			3	4	3		
4	2				11	1	
5							
6							

Clone 7 — t=8 hrs + tet — total cells counted = 488, total abnormal cells = 146 (29.9%), of which 47 (9.6%) were unclassifiable

number of nuclei	number of kinetoplasts						
	0	1	2	3	4	5	6
0		6	2				
1							
2	3	29		10	7		
3		1	5	2	5	1	
4			3	8	15		1
5						1	
6							

Clone 7 — t=10 hrs + tet — total cells counted = 409, total abnormal cells = 208 (50.9%), of which 54 (13.2%) were unclassifiable

number of nuclei	number of kinetoplasts						
	0	1	2	3	4	5	6
0		6	1				
1	3			2			
2	2	31		23	8		
3		1	11	16	2		
4	2	3	5	16	15		
5			1	3	2		
6							
7				1			

Clone 15 t=6 hrs + tet total cells counted = 412, total abnormal cells = 80 (19.4%), of which 48 (11.7%) were unclassifiable

		number of kinetoplasts						
		0	1	2	3	4	5	6
**number of nuclei**	0		2					
	1	2			1			
	2		7		5	10		
	3			1	2	1		
	4				1	1		
	5							
	6							

Clone 15 t=8 hrs + tet total cells counted = 417, total abnormal cells = 133 (31.9%), of which 86 (20.6%) were unclassifiable

		number of kinetoplasts						
		0	1	2	3	4	5	6
**number of nuclei**	0							
	1	5						
	2		15		6	3		
	3			2	8	4		
	4				1	3		
	5							
	6							
